# Management of men with AZFc deletions

**DOI:** 10.1186/s12610-023-00217-8

**Published:** 2024-01-11

**Authors:** Peter N Schlegel

**Affiliations:** Department of Urology, Weill Cornell Medicine, Starr 916A 525 East 68th Street, New York, NY 10065 USA

**To the Editor:**

The published article by Deng et al. [1] confirms a number of concepts that have been previously published regarding management of men with non-obstructive azoospermia (NOA) associated with AZFc deletions. A critical point of their manuscript is the demonstration of the poor predictive value of fine needle aspiration (FNA) mapping for detection of sperm within the testes of men with NOA. For men with negative FNA maps (no sperm seen), sperm were able to be found and used for assisted reproductive using the more effective microTESE (testicular sperm extraction) approach for sperm retrieval in 65% of men. This raises the question of the clinical utility of FNA mapping in management of men with NOA. In essence, why would you ever use FNA mapping for the management of non-obstructive azoospermia?

Prior meta-analyses of comparative trials have demonstrated that FNA is twofold less likely to find sperm in NOA than standard multi-biopsy approaches, and microTESE is 1.5-fold more effective at finding sperm than the multi-biopsy approach [2]. Given the high effectiveness of microTESE, there appears to be no situation when you would consider the FNA map – as its results would lead to microTESE, whether the map showed sperm or not. As noted in ASRM/AUA guidelines [3], microTESE remains the gold standard – the most effective and arguably safest approach for sperm retrieval – in NOA.

Several other facets of the management of men with AZFc deletions are important to highlight. Overall, the ability to get sperm from these men is quite high, relative to other etiologies of (or even idiopathic) NOA. This manuscript only focused on men with azoospermia. But, of particular importance to consider is the relative frequency of cryptozoospermia in men with AZFc deletions. We have observed that nearly 70% of men with AZFc deletions will have rare sperm in the ejaculate. This has led us to perform a careful semen analysis, including the potential evaluation of several aliquots of the centrifuged semen specimen on the day of planned sperm retrieval to avoid unnecessary surgery for these men who can be effectively treated with ejaculated sperm. We have even found and successfully used sperm from the ejaculate in men with AZFc deletions and prior failed biopsy retrieval of sperm.

In this article, Deng et al. have reported on those men with AZFc deletions and azoospermia, but it is important to consider the possibility of having rare sperm identified in the ejaculate for AZFc-deleted patients. In a recent report [4], we have found that for unselected men with NOA, 9% or more of men with prior documented azoospermia can be found to have rare sperm in the ejaculate – obviating the need for planned surgery – if semen analysis is repeated on the day of sperm retrieval.

Deng et al. are to be congratulated for bringing together data on management of men with AZFc deletions and NOA. These observations are a valuable contribution to published literature.

## Data Availability

The data and materials referenced are from published sources and/or are stated in the Letter.
